# The genetic and epigenetic landscapes of the epithelium in asthma

**DOI:** 10.1186/s12931-016-0434-4

**Published:** 2016-09-22

**Authors:** Fatemeh Moheimani, Alan C-Y Hsu, Andrew T Reid, Teresa Williams, Anthony Kicic, Stephen M. Stick, Philip M. Hansbro, Peter A.B. Wark, Darryl A. Knight

**Affiliations:** 1 School of Biomedical Sciences and Pharmacy, Faculty of Health and Medicine, HMRI building, The University of Newcastle, Callaghan, NSW 2308 Australia; 2 Priority Research Centre for Healthy Lungs, Hunter Medical Research Institute, The University of Newcastle, New South Wales, Australia; 3 Department of Biochemistry and Microbiology, University of Victoria, Victoria, Canada; 4 Telethon Kids Institute, Centre for Health Research, The University of Western Australia, Nedlands, 6009 Western Australia Australia; 5 Department of Respiratory Medicine, Princess Margaret Hospital for Children, Perth, 6001 Western Australia Australia; 6 School of Paediatrics and Child Health, The University of Western Australia, Nedlands, 6009 Western Australia Australia; 7 Centre for Cell Therapy and Regenerative Medicine, School of Medicine and Pharmacology, The University of Western Australia, Nedlands, 6009 Western Australia Australia; 8 Department of Respiratory and Sleep Medicine, John Hunter Hospital, New South Wales, Australia; 9 Department of Anesthesiology, Pharmacology and Therapeutics, University of British Columbia, Vancouver, Canada

**Keywords:** Epithelial cells, Asthma, Genes, DNA methylation, Histone acetylation, microRNA

## Abstract

Asthma is a global health problem with increasing prevalence. The airway epithelium is the initial barrier against inhaled noxious agents or aeroallergens. In asthma, the airway epithelium suffers from structural and functional abnormalities and as such, is more susceptible to normally innocuous environmental stimuli. The epithelial structural and functional impairments are now recognised as a significant contributing factor to asthma pathogenesis. Both genetic and environmental risk factors play important roles in the development of asthma with an increasing number of genes associated with asthma susceptibility being expressed in airway epithelium. Epigenetic factors that regulate airway epithelial structure and function are also an attractive area for assessment of susceptibility to asthma. In this review we provide a comprehensive discussion on genetic factors; from using linkage designs and candidate gene association studies to genome-wide association studies and whole genome sequencing, and epigenetic factors; DNA methylation, histone modifications, and non-coding RNAs (especially microRNAs), in airway epithelial cells that are functionally associated with asthma pathogenesis. Our aims were to introduce potential predictors or therapeutic targets for asthma in airway epithelium. Overall, we found very small overlap in asthma susceptibility genes identified with different technologies. Some potential biomarkers are *IRAKM*, *PCDH1*, *ORMDL3*/*GSDMB*, *IL*-*33*, *CDHR3* and *CST1* in airway epithelial cells. Recent studies on epigenetic regulatory factors have further provided novel insights to the field, particularly their effect on regulation of some of the asthma susceptibility genes (e.g. methylation of *ADAM33*). Among the epigenetic regulatory mechanisms, microRNA networks have been shown to regulate a major portion of post-transcriptional gene regulation. Particularly, miR-19a may have some therapeutic potential.

## Background

Asthma affects people of all ethnicities and ages and there has been a substantial increase in the prevalence of asthma over the past few decades, with current estimates of approximately 300 million people suffering from the disease worldwide [1]. Asthma is characterised by coughing, shortness of breath, chest tightness and wheezing, often triggered by exposure to allergens and foreign pathogens [1]. The initial response consists primarily of airway smooth muscle constriction and airway inflammation (oedema, inflammatory cell infiltration, increased airway secretions). Whereas more chronic responses such as structural remodelling of the airway including smooth muscle and sub-mucosal gland hyperplasia and hypertrophy, extracellular matrix (ECM) deposition and angiogenesis are generally thought to occur in parallel with inflammatory responses [1].

The airway epithelium is the interface between the respirable environment and the sub-mucosa and acts as the first defence line against inhaled noxious agents and aeroallergens [2]. The epithelium of conducting airways is pseudo-stratified and consists of ciliated columnar epithelial cells, goblet cells, intermediate columnar epithelial cells, side population cells, serous cells, and basal cells [3]. The epithelium of asthmatics presents several structural and functional abnormalities, including a greater proportion of resident stem cells and basal cells, goblet-cell hyperplasia and excessive mucus production as well as fewer ciliated cells compared to healthy individuals, suggesting dysregulated differentiation [4, 5]. The epithelial abnormalities are associated with increased susceptibility to oxidant-induced stress, aberrant cytokine and ECM release [6], mitotic dyssynchrony [7] and a deficient innate immune response [8–10]. These abnormalities also affect epithelial repair and regeneration processes after injury, leading to defective maintenance of the epithelial barrier and its normal function [2, 11, 12].

Both genetic susceptibility and environmental risk factors influence asthma [13]. Genetic studies have progressed from using linkage designs and candidate gene association studies to genome-wide association studies (GWAS) [14] and whole genome sequencing (WGS) [15], and detected different genes associated with asthma susceptibility. Several of the recent GWAS have shown to have a significant epithelial contribution, which also segregates away from allergy/atopy. Furthermore, different environmental challenges (such as smoking, air pollution, and microbial exposures) can affect gene expression through epigenetic regulation. Epigenetic factors are an important regulator of gene transcription, that do not influence gene sequence [16]. Epigenetic mechanisms include DNA methylation, histone modifications, and regulation by non-coding RNAs, especially microRNAs (miRNAs). This review will focus on identifying genetic and epigenetic candidates in airway epithelium, which are functionally associated with asthma and may act as predictors or therapeutic targets.

### Asthma susceptibility genes in airway epithelium

Early genetic studies relied on positional cloning in combination with linkage analysis leading to detection of genes associated with asthma expressed in airway epithelium including A disintegrin and metalloprotease 33 (*ADAM33*), *GPRA*, protocadherin-1 (*PCDH1*), Serine protease inhibitor Kazal type-5 (*SPINK5*), IL-1 receptor associated kinase-M (*IRAKM*), Dipeptidyl-peptidase 10 (*DPP10*) and *HLA*-*G* genes (Table 1) [17–31]. *ADAM33* on chromosome 20p13, was the first asthma susceptibility gene discovered [17]. ADAM33 protein is expressed in many cells including the airway epithelium [18], fibroblasts and smooth muscle cells [17, 18, 32] and is known as a membrane-anchored metalloprotease with diverse functions, including shedding of cell-surface proteins such as cytokines and cytokine receptors [17]. *ADAM33* has be associated with airway remodelling and bronchial hyperresponsiveness (BHR) through epithelial–mesenchymal trophic unit (EMTU), leading to proliferation of biosynthetically active fibroblasts, myofibroblasts and smooth muscle [17]. *PCDH1* is located on chromosome 5q31-q33 and encodes the protocadherin-1 protein [22, 23]. The expression of PCDH1 is aligned with the apical adhesion complex expression in airway epithelial cells hence association of *PCDH1* with asthma is proposed to be through epithelial structural defects leading to BHR [22, 23] and is IgE independent [24]. Dysregulation of PCDH1 expression in asthma also leads to impaired differentiation of epithelial cells [23]. Another gene *is DPP10* which shown to preferentially expressed in the epithelium of asthmatics [27]. *DPP10*, is located on 2q14-32 and encodes the di-peptidyl peptidase like 10 protein, which unlike other members of DPP family is unable to cleave the terminal of dipeptides from cytokines and chemokines [27, 28]. These suggest other potential mechanisms for *DPP10* association with asthma. In the nervous system, DPP10 has been shown to modulate the electrophysiological properties, cell-surface expression and subcellular localisation of voltage-gated potassium channels [33]. Considering the important role of potassium ion channels in asthma [34], DPP10 may also be involved in this process although this requires further investigation. Furthermore, Zhou et al. reported the association of *DPP10* with BHR in Chinese population [29]. *HLA*-*G* on chromosome 6p21 is also expressed highly in bronchial epithelial cells of asthmatics and is associated with BHR [30]. HLA-G inhibits the effecter function of T cells and natural killer (NK) cells [35]. Three miRNAs; miR-148a, miR-148b, and miR-152 have been reported to affect *HLA*-*G* expression, suggesting that miRNA mediated mechanisms may contribute to the impact of *HLA*-*G* on asthma risk [31].

**Table 1:** Asthma susceptibility genes identified by positional cloning and genome-wide association (GWAS) in airway epithelium

Positional cloning				
Chromosome		Gene	Function	Reference
20p13		ADAM33	Airway remodelling and BHR	[17]
5q31-q33		PCDH1^a^	Airway remodelling and BHR	[22–24]
2q14-32		DPP10	BHR	[27–29]
6p21		HLA-G	BHR	[30, 31]
7p15-p14		GPRA/NPSR1/GPR154	Cell homeostasis	[19–21]
5q31-35		SPINK5/LEKTI	Protective against allergen/inflammation	[25]
12q13-24		IRAKM^b^	Inflammation	[26]
Genome-wide association (GWAS) and associated SNP				
Chromosome	SNP	Gene	Function	References
2	rs3771166	IL1RL1 and IL18R1	Alarmin to alert the immune system after epithelial cell damage during trauma or infection	[45, 50]
6	re9273349	HLA-DQ	Recognition of non-self antigens	[13]
9	rs1342326	IL33+	Alarmin	[13, 50]
15	rs744910	SMAD3	TGF-β1 signaling and response to respiratory viral infection	[50, 57–59]
17	rs2305480	ORMDL3^b^+ GSDMB^b^+	BHR Epithelial cell homeostasis	[13, 64, 66–68]
5	rs1837253	TSLP+	Epithelial cell homeostasis and improving wound healing-Protective role against asthma	[42, 43, 71–74]

^a^Adult and children

^b^Early onset

+common between different ethnic group

Other studies detected *GPRA* (also known as Neuropeptide S Receptor 1; *NPSR1*, and *GPR154*) on chromosome 7p15-p14 [19–21]. Both GPRA, which belongs to the G protein-coupled receptor family, and its agonist, Neuropeptide S (NPS) are co-expressed in bronchial epithelium and specific activation of the GPRA-A isoform with NPS inhibits cell growth [19, 20]. Since the balance between epithelial cell proliferation and regeneration is dysregulated in asthmatics [4, 36], *GPRA* likely plays an important role in the pathogenesis of disease [19, 20]. Further studies identified the *SPINK5* gene on chromosome 5q31-35 which encodes a multidomain serine protease inhibitor known as lympho-epithelial Kazal-type-related inhibitor (LEKTI). LEKTI has been shown to be a major physiological inhibitor of multiple serine proteinases, including the exogenous serine proteases trypsin, plasmin, subtilisin A, cathepsin G and neutrophil elastase [37]. *SPINK5* is essential in the epidermal barrier function through regulating protease activity [38] and LEKTI plays a crucial role in skin homeostasis by selectively inhibiting human kallikrein-related peptidase genes including, KLK5, KLK7 and KLK14 [39]. LEKTI may therefore protect the epithelium against allergens or inflammatory related proteases. However, the exact function of *SPINK5* in airway epithelium remains to be elucidated. Another asthma susceptibly gene is *IRAKM*, which is located on chromosome 12q13-24. IRAK-M regulates NF-kB and inflammation via suppressing Toll-like receptor/IL-1R pathways. When IRAK-M function is hampered, overproduction of inflammatory cytokines in the lung in response to infection/allergens may result in a Th2-mediated allergic response and/or Th1-dependent exacerbation of asthma symptoms [26].

Further technological advances led to GWAS [40], and associated single nucleotide polymorphisms (SNPs) [13], which detected a completely different set of genes; interleukin (IL) 1 receptor-like 1 (*IL1RL1*) and IL18 receptor 1 (*IL18R1*), *IL33*, *HLA*-*DQ*, *SMAD3*, thymic stromal lymphopoietin (*TSLP*), ORM1-like 3 (*ORMDL3*) and gasdermin B (*GSDMB*) as asthma susceptibility genes expressed in airway epithelium (Table 1) [13, 14, 41–43]. *IL1RL1* and *IL18R1* contain SNP rs3771166 on chromosome 2 [13, 44]. IL1RL1 (also known as T1, ST2, DER4, or FIT-1) belongs to the IL-1 superfamily and is the receptor for IL-33 [45]. *IL33* with SNP rs1342326 located on chromosome 9 is also associated with atopic asthma [13, 46, 47]. IL-33 possesses potent transcriptional-repressive properties and is constitutively expressed in epithelial cells [48]. It has been shown that IL-33 activates NF-kB and mitogen-activated protein (MAP) kinases, and induces production of T-helper (Th) 2-associated cytokines, including IL-4, IL-5, and IL-13 [49]. In this context, IL-33 functions as a prototypical ‘alarmin’ and an endogenous ‘danger’ signal to alert the immune system after epithelial cell damage during trauma or infection [50] and plays an essential role in pro-inflammatory pathway in asthma [13]. *IL18R1* encodes the receptor for IL-18 [45]. IL-18 modulates innate and adaptive immune responses by increasing interferon (IFN)-γ production by Th1 and natural killer (NK) cells or by activating IgE production and Th2 cell differentiation [45].

Another candidate gene detected by GWAS is *HLA*-*DQ* region of the major histocompatibility (MHC) gene located on chromosome 6, which contains SNP rs9273349 [51]. The airway epithelium expresses MHC class II; a heterodimer molecule that consists of an α- and a β-chain in one of three HLA loci: DR, DP and DQ [52], on their surface [53]. Immune response to allergens is also related to specific HLA-DR and DQ haplotypes [13], and is associated with asthma induced by house dust mite, aspirin, soybean, and occupational triggers [54]. However, the exact role of *HLA*-*DQ* in airway epithelium still remains unclear.

*SMAD3*, with SNP rs744910 located on chromosome 15, is another asthma susceptibility gene [13]. SMAD3 is an essential signal transducer in transforming growth factor (TGF)-β signalling, which is elevated in airway epithelial cells of some asthmatics [55]. TGF-β1 induces epithelial–mesenchymal transition (EMT) in airway epithelial cells via a SMAD3-dependent transcription factor snail1 (SNAI1) which transcriptionally supresses E-cadherin [36, 56]. Furthermore, the TGF-β/SMAD3 pathway play essential roles in the airway epithelial response to respiratory viral infection [57–59], including increasing replication of both respiratory syncytial virus [57, 58] and rhinovirus [59].

Among asthma susceptibility genes, *ORMDL3* and *GSDMB*, with SNP rs2305480 at chromosome 17q21, are associated with childhood asthma [60]. *ORMDL3* is a member of a gene family that encodes transmembrane proteins anchored in the endoplasmic reticulum of airway epithelial cells, predominantly [60, 61]. Allergens induce *ORMDL3* expression in airway epithelium leading to increased expression of asthma-associated chemokines, metalloproteases and the unfolded protein response (UPR), which may implicate the potential link between *ORMDL3* and asthma [61]. *ORMDL* regulates ORM protein expression in airway epithelial cells, which is induced in response to allergen challenge [62]. ORM proteins are important homeostatic regulators of sphingolipid metabolism [63], which is associated with the pathogenesis of asthma [64]. Sphingolipids are pivotal in maintenance of cell structure and signaling pathways in physiological and pathological processes; e.g. proliferation, apoptosis and migration [63, 65] and have been shown to contribute to BHR in experimental models of asthma [66]. Further meta-analysis has showed that SNP rs7216389 in the *ORMDL3* may play essential and independent predisposing roles in ethnically diverse populations for both childhood and adult-onset asthma [41]. *GSDMB* is adjacent to *ORMDL3* and is a member of gasdermin family that encodes gasdermin B protein which has roles in secretory pathways, epithelial cell differentiation, cell cycle control and apoptosis [67, 68]. Furthermore, there are several response elements for interferon regulatory factors present in the *GSDMB* promoter region and epithelial interferon-α induces *GSDMB* gene and protein in human nasal epithelial cells, in vitro [69]. *GSDMB* has been proposed to be the causative gene associated with asthma [70].

Among the candidate genes identified by GWAS, *TSLP* on chromosome 5 plays protective roles against the risk of asthma, atopic asthma and BHR across various ethnic groups [42, 43, 71–73]. The rs1837253 SNP may be directly involved in the regulation of TSLP secretion in primary nasal epithelial cells [42]. TSLP is an IL-7 like cytokine that induces myeloid dendritic cells to stimulate the differentiation of naive CD4^+^ T cells to Th2 cells. TSLP mRNA and protein are highly expressed in the asthmatic airway epithelium [72–74]. TSLP has been shown to induce bronchial epithelial cell proliferation and increases repair responses to injury through IL-13 production [74].

Collectively these genes are important in epithelial cell damage, innate and adaptive immunity, and airway inflammation, which are pivotal in the pathology of asthma. Furthermore, some of the products associated with these genes can determine the phenotype of asthma. For instance, the level of IL-33 is highly elevated and widely distributed in bronchial epithelial cells of moderate and severe asthmatics [48]. IL-18 may also contribute to asthma exacerbations in mild and moderate asthmatics through activation of immunologic responses [51]. Given the relationship to asthma endotypes, these genes may indicate pathways for therapeutic intervention. In fact, Phase II trials are currently proceeding using an anti-TSLP antibody; AMG 157 from Amgen Corp., to neutralise the TSLP cytokine for the treatment of allergic diseases as asthma [75].

Notably, only a few genes, such as *IL33* and *TSLP*, are shared among all asthmatics [42, 76, 77] and may play roles as potential biomarkers. Furthermore, while the association of the 17q21 locus (*ORMD*/*GSDMB*) with asthma is the most consistent finding from different studies, there is limited evidence to validate certain SNPs [14]. Integrative genomics defined as identification of causal genes and variants, with improved statistical power, is a promising new approach. By using gene expression as a phenotype and examining how DNA polymorphisms contribute to both gene expression (expression quantitative trait loci; eQTLs) and disease phenotypes, true causal relationships can be discovered [78–80]. Although GWAS have identified loci that are strongly associated with asthma, the molecular mechanisms underlying these associations rely on other technology such as eQTLs [78].

One eQTL study showed that chromosome 17q21, which contained strong GWAS hits, also regulates expression levels of cyclin-dependent kinase 12 (*CDK12*), protein phosphatase 1 regulatory subunit 1B (*PPP1R1B*), titin-cap (*TCAP*) and StAR-related lipid transfer (*START*) domain containing 3 (*STARD3*) genes in the airway epithelium [78]. CDK12 is a member of the cyclin-dependent kinase (CDK) family, which are serine/threonine kinases regulating cell cycle progression [78, 81]. Airway epithelial cells from asthmatics overexpress the CDK inhibitor; p21^waf^ [82], which may explain the abnormal repair responses of the airway epithelium of asthmatics after wounding [82]. However, the role of *TCAP*, *PPP1R1B*, *STARD3* in asthma are still unknown [78]. Furthermore, epithelial eQTL detected Cystatin SN (*CST1*) on chromosome 20p11.21, which contains SNP rs16856186 [78]. CST1 may neutralise cystatin C; a potent cathepsin B inhibitor, and increase cell proliferation [83]. *CST1* is expressed differentially in airway cells of asthmatics with exercise-induced bronchoconstriction (EIB) compared to asthmatics without EIB [84]. eQTL also confirmed cadherin-related family member 3 (*CDHR3*) gene, as an epithelial susceptibly gene for severe exacerbations in childhood asthma [78, 85]. *CDHR3* encodes a hemophilic cell adhesion molecule, which may be involved in maintaining cell integrity by forming cell-cell junctions. Furthermore, functional disruption of *CDHR3* has been reported in human rhinovirus-induced asthma exacerbation [86]. Also, epithelial eQTL supported *SPINK5* as an asthma susceptibility gene [86], as described earlier.

It is also essential to note that in a disease as complex as asthma, it is unlikely that one or a few functional gene variants will be responsible for all pathophysiological events. While GWAS have been useful and continue to identify novel genes for allergic diseases through increased sample sizes and phenotype refinement, further approaches to integrate analyses of rare variants, eQTL approaches, and epigenetic mechanisms will likely lead to greater insight into the genetic basis of the disease.

The advent of whole genome sequencing (WGS), which includes copy number variants (CNVs) and low-frequency variants, has been proposed to overcome the drawbacks of the earlier technologies [15, 76]. CNVs, which are genetic variants including the deletion or duplication of more than 50 bp of gene sequence [15], are one the most recent advances to detect asthma susceptibility genes. Recently, an association between a 6 kbp deletion in an intron of *NEDD4L* with increased risk of asthma was reported but only in Hutterites [15]. *NEDD4L* is expressed in bronchial epithelial cells, and *NEDD4L* knockout mice showed severe airway inflammation and mucus accumulation [15].

To adequately assess the entire genome, a large number of genetic polymorphisms (250,000 to 1 million) is required and the number of polymorphisms will vary between studies due to different levels of linkage disequilibrium [14]. Currently, WGS is neither affordable nor feasible on the large number of individuals to acquire sufficient power for detecting associations with asthma [15].

### Effect of environmental exposure on asthma

Environmental factors play essential roles in asthma aetiology. The increase in the prevalence of asthma worldwide during recent decades, the substantial variations in populations with a similar racial and ethnic background but exposed to different environmental stimuli, and the significant increase in the frequency of occupational asthma are all pointing out toward the important role of environmental factors [87].

Environmental stimuli affecting asthma are categorised to outdoor and indoor factors. Outdoor stimuli that trigger or exacerbate asthma include microbial and viral pathogens, airborne particulates, ozone, diesel exhaust particles, pollens, outdoor moulds, environmental tobacco smoke, cold air, and humidity [87, 88]. Indoor environmental factors include allergens derived from dust mites, cockroaches, mice and pets which has been shown to induce airway inflammation; particles generated from indoor burning of tobacco, wood, and biomass; and biological agents such as indoor endotoxin, products from gram-positive bacteria, and 1,3-β-glucans from moulds [87, 88].

In particular relation to asthma susceptibility, the exposure to specific environmental factors can play key factor in the induction or suppression of asthma-related genes. The main areas of studies in regards to the impact of gene-environment interactions on asthma development and pathogenesis have been so far related to smoking, air pollution, and microbial exposures. Maternal smoking is one of the major risk factor for asthma in offspring. Maternal smoking substantially enhances the strength of the linkage signal on chromosome 5q31–34 to asthma in the children [89, 90]. Furthermore, polymorphic variation in candidate genes known to be involved in asthma, for example *TNF*-*308* and *glutathione*-*S*-*transferase M1* (*GSTM1*; involved in detoxification of oxidative stress and lung function growth in children), are predictors of BHR to passive smoking [89, 91]. The most well-known interaction between environmental factors and gene is between endotoxin with Toll like receptor (TLR)-4 with further impact on adaptive immune response, epithelial and smooth muscle cells through NF-kB. Polymorphism in TLR-4 is related to asthma and it is proposed that the other TLRs (e.g. TLR-9 and -3) present the similar polymorphic associations with other environmental stimuli, such as CpG methylation of TLR-9 and double-stranded RNA (dsRNA) for TLR-3 [89, 92]. These reports point out to the importance of early life environmental factors, such as passive smoking, pollutant exposure and viral infections, as a perverse factor on the developing asthma in childhood.

However little is known about the effect of environmental-gene interactions in airway epithelium of asthmatics. It has been shown that particulate matter with a diameter of <10 μm diameter (known as PM10) increases HAT activity and the level of acetylated histone 4 (H4) through oxidative stress. PM10 induced histone acetylation is associated with promoter region of the *IL*-*8* resulting in increased IL-8 gene and protein release from alveolar epithelial (A549) cells [93]. Interestingly, butyrate; a fermentation product of intestinal bacteria, also showed to enhance histone acetylation by inhibition of HDAC enzymes leading to an increase in gene expression of inflammatory cytokines in intestinal epithelial cells [94]. Cigarette smoke-induced oxidative stress also reduces HDAC2 and increases cytokines expression in alveolar macrophages [95] but the effect on airway epithelium is yet to be determined.

Most importantly, many of the indoor and outdoor asthma triggers also have demonstrable reprogramming effects on the immature airway during early life, leading to altered asthma risk in later life. Asthma hence is not a homogeneous disease but a condition influenced by interactions between genetic and environmental factors through epigenetic mechanisms that influence gene expression.

### Epigenetic regulatory factors in airway epithelium

**Fig. 1:**
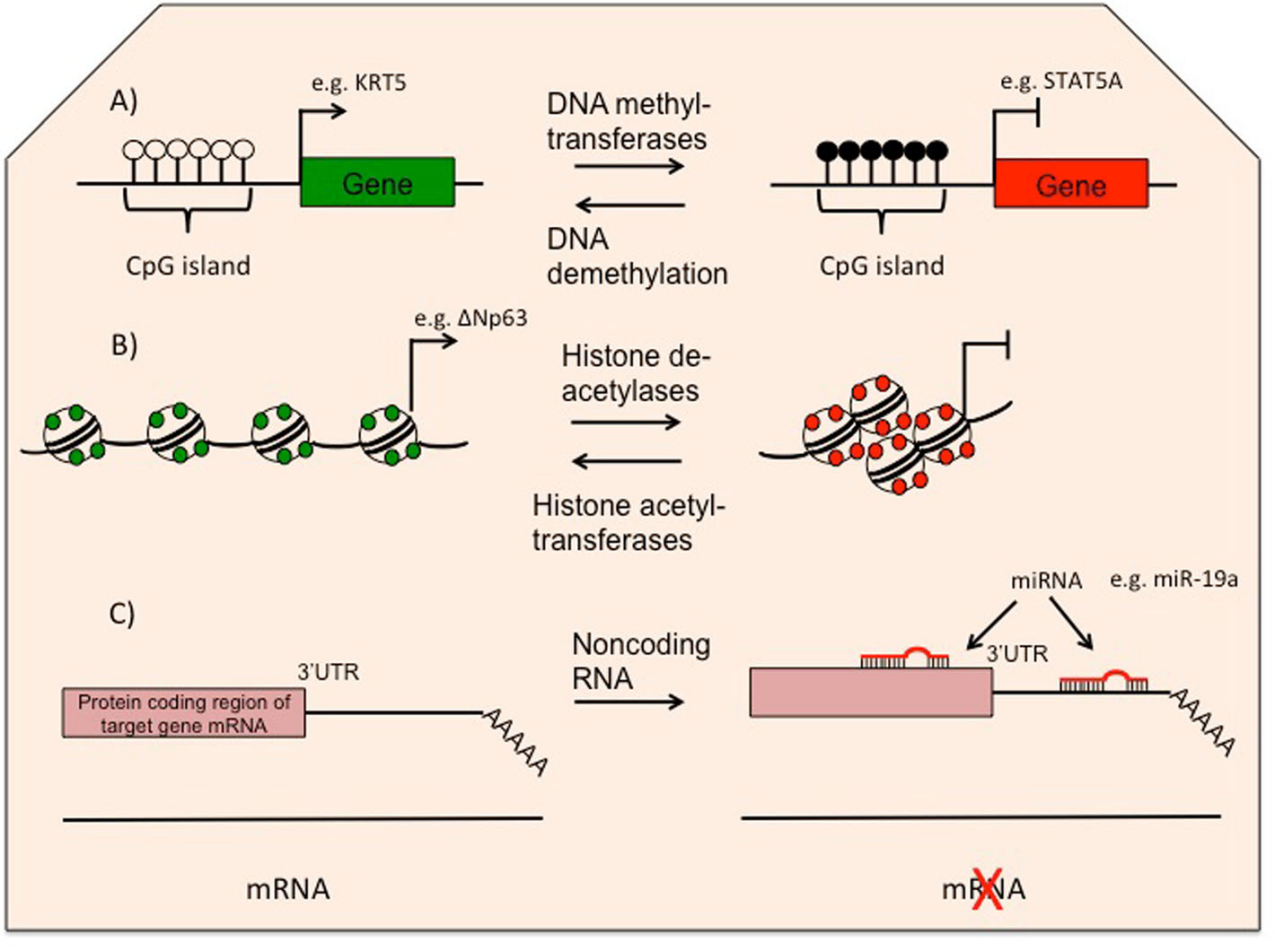
Epigenetic regulatory factors in airway epithelium. **a** DNA methylation; *white circles* represent unmethylated CpGs that induces gene expression (e.g. KRT5) while *black circles* represent methylated CpGs that suppresses gene expression (e.g. STAT5A). **b** Histone acetylation; *green circles* refer to acetylated histone tail that stimulate gene expression (e.g. ΔNp63) while *red circles* indicate free histone tails that suppressed gene expression. **c** Noncoding RNA; miRNAs affect gene expression by either RNA degradation or translational inhibition. miRNAs hence (e.g. miR-19a) may suppress mRNA expression (e.g. TGF-β receptor 2)

Environmental challenges can affect gene expression through epigenetic mechanisms. Epigenetics is described as a heritable regulation of gene transcription that does not require alterations in gene sequence [16]. Epigenetic changes may form stable heritable changes in gene expression and in a tissue-specific fashion [96, 97]. Particularly, epigenetic regulation affects gene expression through three main mechanisms, including DNA modifications, histone modifications, and non-coding RNAs (Fig. 1 and Table 2).

**Table 2:** Epigenetic regulatory factors associated with asthma in airway epithelial cells

DNA modification signatures			
Gene	Status	Function	References
KRT5^a^	Hypo-methylation	Epithelial homeostasis	[97, 99]
STAT5A^a^	Hyper-methylation	Immune system, Cell proliferation	[99]
CRIP1^a^	Hyper-methylation	Epithelial homeostasis, transcription	[99]
ARG2^a^	Hyper-methylation	Reduced FeNO	[100]
IL-6^a^	Hypo-methylation	Increased FeNO	[98]
iNOS^a^	Hypo-methylation	Increased FeNO	[98]
ADAM33	Hyper-methylation	BHR	[101]
Histone modification signatures			
HDAC/HAT	Status	Function	References
H3K18	Acetylation	Increases the expression of ΔNp63, EGFR and STAT6 affecting epithelial homeostasis	[109]
HDAC2^a^	De-acetylation	Anti-inflammatory	[112]
miRNA signatures			
miRNA	Status	Function	References
let-7f^b^	Overexpressed	unknown	[121]
miR-487b^b^			
miR-181c^b^			
miR-203^b^	Suppressed	Targeting p63 and c-Abl	[121–123]
miR-34/449 family	Suppressed	Targeting NOTCH1 mRNA and affecting cell homeostasis	[125]
miR-18a	Suppressed	activation/signalling of IL-6 and IL-8	[129]
miR-27a			
miR-128			
miR-155			
miR-19a^c^	Overexpressed	Targeting TGF-β receptor 2 mRNA and affecting cell homeostasis	[130]

^a^Children

^b^Mild asthma

^c^Severe asthma

#### DNA modifications

Modification of DNA occurs through addition or removal of small covalent molecules such as methyl or acetyl groups. DNA methylation occurs when methyl groups are added by a DNA methyltransferase (DNMT), onto cytosine nucleotides that are followed by guanine residues (CpG sites). Gene promoters are relatively rich in CpG sites which are known as CpG islands. DNA methylation can lead to gene expression silencing through formation of 5-methyl-cytosine (5mC) [98].

Recent studies have characterised a number of DNA methylation signatures in epithelial cells of asthmatics, including cytokeratin 5 (*KRT5*) [99], signal transducer and activator of transcription 5A (*STAT5A*) [99], cysteine-rich protein 1 (*CRIP1*) [99], arginase 2 (*ARG2*) [100], *IL*-*6* [98], inducible nitric oxide synthase (*iNOS*) [98] and *ADAM33* [101] (Table 2).

KRT5 is a marker of basal cells and its expression is increased in the epithelium of asthmatics [5, 36]. In asthmatic children, *KRT5* exhibits reduced methylation resulting in increased expression of this gene [97, 99]. Increased KRT5 may hence be associated with dysregulated epithelium differentiation. The STAT5A transcription factor is activated by different pro-Th2 cytokines (e.g. IL2, IL7, or TSLP) suggesting its significant role in promoting Th2 cell differentiation and responses [102] and epithelial cell proliferation [103]. CRIP1 has been reported to play a role in cell motility, adhesion, and structure through interaction with the cytoskeletal protein actin [104] and also translocates to the nucleus to facilitate protein interactions important for transcriptional regulation [105]. The promoters of *STAT5A* and *CRIP1* are hyper-methylated in epithelium of asthmatic children [99], resulting in decreased expression of STAT5A, contrary to increased CRIP1 expression [99]. Further studies are therefore needed to understand the roles of *STAT5A* and *CRIP1* in epithelial function.

*ARG2*, *IL*-*6* and *iNOS* are three methylated genes that have been related to fractional exhalation of nitric oxide (FeNO) in asthmatic children [98, 100, 106]. Methylation of the *ARG2* promoter in asthmatic children is associated with reduced FeNO [100]. However, asthmatic children with lower DNA methylation of the *IL*-*6* and *iNOS* promoters in nasal epithelial cells had higher airway inflammation, as measured by increased FeNO [98]. Therefore, further investigation is required to determine the underlying biological mechanisms driving the association of these DNA methylations with FeNO and whether children with different degrees of asthma severity and symptom management have different levels of DNA methylation.

Furthermore, hyper-methylation on *ADAM33* in bronchial epithelial cells is strongly associated with BHR, irrespective of asthma status [101]. This is in contrast to *ADAM33* hypo-methylation in fibroblasts, which is speculated to be involved in airway remodelling [107]. This highlights the importance of cell specific epigenetic changes, as well as the potential challenges in developing novel therapeutics.

Recently, DNA methylation has been shown to occur in airway epithelial cells isolated from asthmatics after a single 24 h exposure to IL-13 [108]. Intriguingly, areas of methylation were mainly adjacent to asthma susceptibility genes and in particular genes related to fibrotic and inflammatory pathways (e.g. neutrophil cytosolic factor 2; *NCF2*, and *MMP14*) [108]. Global and gene specific methylation status in the airway epithelium however still requires further investigation before potential targets can be identified and trialled as therapies for asthma.

#### Histone modifications

The DNA of each cell is packaged into nucleosomes where its 147 base pairs wrap around an octamer of four core histone (H2A, H2B, H3, and H4). The covalent alterations of the amino acid residues of core histone N-terminal tails are essential for modification of the chromatin structure and regulate gene expression. For example, the acetylation of lysine residues on histone tails via histone acetyltransferases (HATs), generally results in increased gene transcription whereas removal of the acetyl group via deacetylases (HDACs) leads to gene suppression. In contrast, methylation of histone tails can be both activating and suppressing depending on the particular residue. Methyl groups are added to lysine or arginine residues by histone methyltransferases (HMTs) and removed by histone demethylases (HDMs) [109, 110].

Increased HAT activity and reduced HDAC activity in biopsies from mild asthmatics have been reported to lead to the increased expression of multiple inflammatory genes [111]. Interestingly, these activities may be partially reversed by treatment with inhaled corticosteroids [111]. Furthermore, within the adult airway epithelium, elevated histone H3 lysine 18 (H3K18) acetylation and histone H3 lysine 9 trimethylation (H3K9me3) have been shown in asthmatics [109]. H3K18 acetylation increases the expression of *ΔNp63*, *EGFR*, and *STAT6*, which, are known to be altered in the epithelium of asthmatics [109]. Very few studies have investigated histone modifications in children with asthma. In one study, passive cigarette smoke reduced HDAC2 activity and protein expression via PI3K signalling in children with severe asthma. This is believed to suppress the anti-inflammatory effects of corticosteroid treatment [112] (Table 2).

Whether histone modifications in epithelial cells are major contributors in conferring asthma susceptibility and/or severity remains to be determined.

#### Non-coding RNAs

A number of classes of noncoding RNAs have been discovered in mammalian cells including long non-coding RNAs (lncRNAs), Piwi-interacting RNAs (piRNAs), and miRNAs.

lncRNAs are non-protein coding RNA transcripts longer than 200 nucleotides. There are approximately 15,000 lncRNAs discovered so far although only a small number of which have been shown to be biologically relevant. piRNAs are small non-coding RNAs of 26–31 nucleotides long, predominantly found in spermatogenic and ovarian cells [113]. Their functions have been linked to both epigenetic and post-transcriptional gene silencing [113]. piRNAs interact with piwi protein, a RNA-binding protein, which degrades target mRNAs to prevent protein translation [113]. There have been no studies that extensively investigate profiles of lncRNAs and piRNAs in airway epithelium of asthmatics. Only one study has shown that piR30840 directly targets and degrades IL-4 mRNAs leading to inhibition of the development of Th2 T-lymphocytes. Furthermore, the level of piR30840 is significantly reduced in serum from patients with asthma [113].

miRNAs are proposed to control expression of 30–60 % of human genes [114] and hence are crucial in most biological and pathological processes including cell proliferation, differentiation, apoptosis, carcinogenesis and immune responses [115–117]. miRNAs are 20–24 nucleotides long and bind to the 3′ untranslated region (UTR) of target mRNAs resulting in their degradation or translational inhibition [118–120]. Currently, there are over 1000 miRNAs identified in miRbase (www.mirbase.org), many of which have multiple binding partners and thus affect multiple pathways [118]. miRNAs may modulate protein synthesis at both initiation and post-initiation of translation [118]. miRNAs have also been shown to up-regulate some mRNA targets [120]. The balance between up- and down-regulation of miRNA plays a pivotal role in cell cycle and process of cell proliferation and regeneration, a process that is dysregulated in asthmatic epithelium. miRNAs are therefore interesting regulatory factors which may contribute substantially to airway epithelium abnormalities in asthmatics.

#### miRNA expression in epithelium of asthmatics

**Fig. 2:**
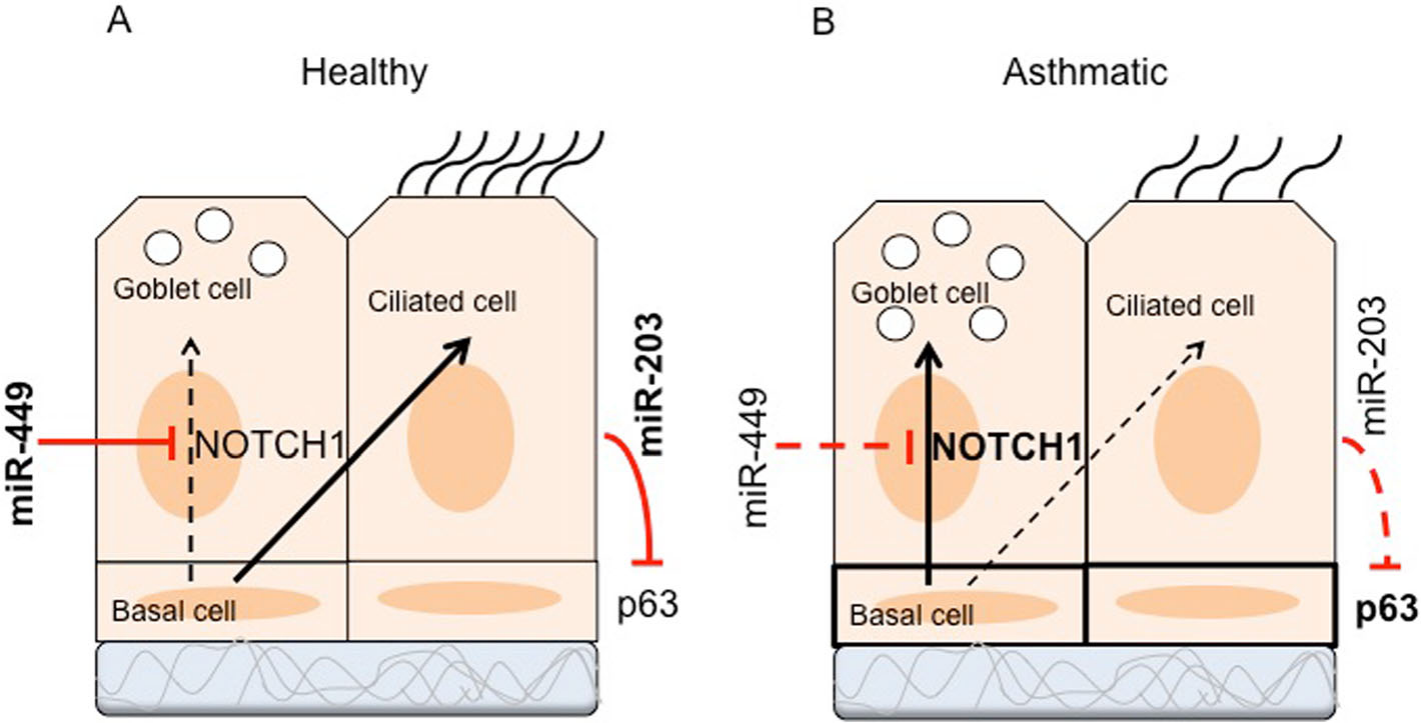
Role of miRNAs in airway epithelial cells regeneration. **a** In healthy airway epithelial cells, miR-449 suppresses NOTCH1 mRNA and encourages differentiation of ciliated cells compared with goblet cells. miR-203 may paly essential role in epithelial cell homeostasis by suppressing p63 which is expressed in basal cells. **b** The level of miR-449 and miR-203 are reduced in asthmatic airway epithelial cells, which may result in increase in goblet cells and compromising epithelial cells regeneration, respectively. *Solid lines* and *bold fonts* represent strong effect and dashed lines and normal font represent weak effect

There are a limited number of studies examining miRNA expression in epithelium of asthmatics or non-asthmatics (Table 2). miRNA microarray performed on primary bronchial epithelial cells cultured at air-liquid interface (ALI) showed higher expression of let-7f, miR-181c* and miR-487b but lower expression of miR-203 in mild asthmatics compared with healthy controls [121]. miR-203 has been shown to play a potent role in the (keratinocyte) self-renewal program during epidermal differentiation by targeting *p63*, facilitating cell cycle exit and promoting differentiation [122]. Given that the epithelium of asthmatics expresses higher levels of p63 [36], it is intriguing to speculate that there is a direct link between miR-203 and p63 (Fig. 2). Recently, miR-203 has also been shown to inhibit airway smooth cell proliferation through targeting the non-receptor tyrosine kinase c-Abl (Abelson tyrosine kinase, Abl, ABL1) [123]. In particular, Abl kinases regulate cell-cell adhesion in epithelial cells and fibroblasts through cadherin-mediated adhesion signals via regulating the activities of the Rac and Rho GTPases [124]. Since, c-Ab1 plays important roles in regulation of the actin cytoskeleton and hence different cellular functions such as proliferation, cell adhesion and migration as well as growth and development [123], the reduced level of miR-203 in epithelial cells of asthmatics may promote cell proliferation, increase goblet cell hyperplasia and/or decrease ciliated cells. Genome wide profiling of bronchial epithelial brushings also revealed four members of the miR-34/449 family (miR-34b-5p, miR-34c-5p, miR-449a, and miR-449b-5p) were significantly suppressed in asthma [125]. Interestingly, no clear relationship was observed between these differentially expressed miRNA and serum IgE level in asthmatics [125], which indicates the role of these miRNAs at cellular and molecular levels rather than inflammatory and allergic responses. Furthermore, inhaled corticosteroids showed only minor effects on miRNA expression, and failed to restore miRNA levels to healthy control levels [125]. The miR-34/449 family are closely associated with regulation of epithelial cell proliferation and differentiation. Specifically, miR-449 is essential in regulation of airway ciliated cells by targeting *NOTCH1* [126]. Notch signaling triggers airway mucous metaplasia and inhibits alveolar development (ciliated cells) [127]. The low level of miR-449 in epithelial cells of asthmatics may therefore shift the fate of these cells toward more mucous production (Fig. 2). Furthermore, miR-34/449-deficient mice suffer from primary ciliary dyskinesia (PCD). These abnormalities have been shown to be mediated by Cp110, a centriolar protein suppressing cilia assembly, that is a target of miR-34/449 [128]. A panel of miRNAs including miR-18a, miR-27a, miR-128 and miR-155 were also down-regulated in the epithelium of asthmatics. These miRNAs are involved in activation/signaling of IL-6 and IL-8 [129].

Recently, miR-19a was reported to be the only miRNA that differentiates severe from mild asthma. miR-19a is up-regulated in severe asthmatic epithelial cells and is not restored by corticosteroids [130]. Elevated levels of miR-19a further stimulate cell proliferation of epithelial cells by targeting TGF-β receptor 2 mRNA. Overexpression of miR-19a results in reduction in phosphorylated SMAD3 whereas suppression of miR-19a facilitates SMAD3 phosphorylation through TGF-β receptor 2 signaling and restore epithelial cells proliferation [130]. These may suggest the potential of miR-19a as a therapeutic target in airway epithelium of asthmatics to re-establish epithelial regeneration.

There are limited numbers of investigations on miRNAs expression in asthmatic epithelium of children, possibly due to difficulty in obtaining epithelial samples. Higher level of miR-148b; a member of miR-152 family, was reported in airway epithelial cells of adults with an asthmatic mother and correlates with sHLA-G levels in the BAL fluid of these subjects [131]. The prenatal effects of maternal asthma on the regulation of foetal genes in airway cells may persist well into adulthood through miRNA regulatory mechanisms.

#### Other miRNAs in airway epithelium with potential role in asthma development

Airway epithelial cells display dysregulted differentiation in asthma. It is therefore important to investigate potential miRNAs fundamental for lung development and epithelial cell homeostasis such as miR-17 family [132, 133]. miR-17 family consist of three paralog clusters of miR-17–92 (miR-17-5p, miR-18, miR-19b, miR-20a, miR-92, miR-19a and miR-17-3p), miR-106a–363 (miR-106a, miR-18b, miR-20b, miR-19b-2, miR-92–2, and miR-363), and miR-106b–25 (miR-106b, miR-93, and miR-25) [134]. Among the miR-17 family, the miR-17-92 cluster and miR-106b have been shown to be essential in maintaining of the structural homeostasis of developing lung epithelium [132, 134]. miR-17-5p, miR-19b and miR-20 are increased in the epithelium and mesenchyme of the embryonic lung compared with fully developed lung [132]. Furthermore, miR-17, miR-20a and miR-106b modulate fibroblast growth factor (FGF)10–FGFR2b signaling by specifically targeting *STAT3* and *MAPK14*, and altering E-cadherin distribution. This is vital for epithelial bud morphogenesis in response to FGF10 signaling [134]. STAT3 and MAPK14 signalling play important roles in airway homeostasis. STAT3 stimulates regeneration and multiciliogenesis by inhibition of the Notch pathway and direct regulation of genes such as *Mcidas* and *Foxj1* [135]. MAPK14 (known also as p38α) has also been shown to regulate lung stem or progenitor cell proliferation and differentiation [136]. MAPK14 regulates C/EBP and HNF3b which are necessary for the differentiation of the stem cells into AT2 and Clara cells, while coordinately suppressing the regulators of stem and progenitor cells proliferation; cyclin D1 and EGFR [136]. These findings emphasise the role of these signalling pathways as well as their regulatory miRNAs in airway epithelial cells homeostasis, which is dysregulated in asthma. These pathways may initiate development and progression of asthma.

## Conclusions

**Fig. 3:**
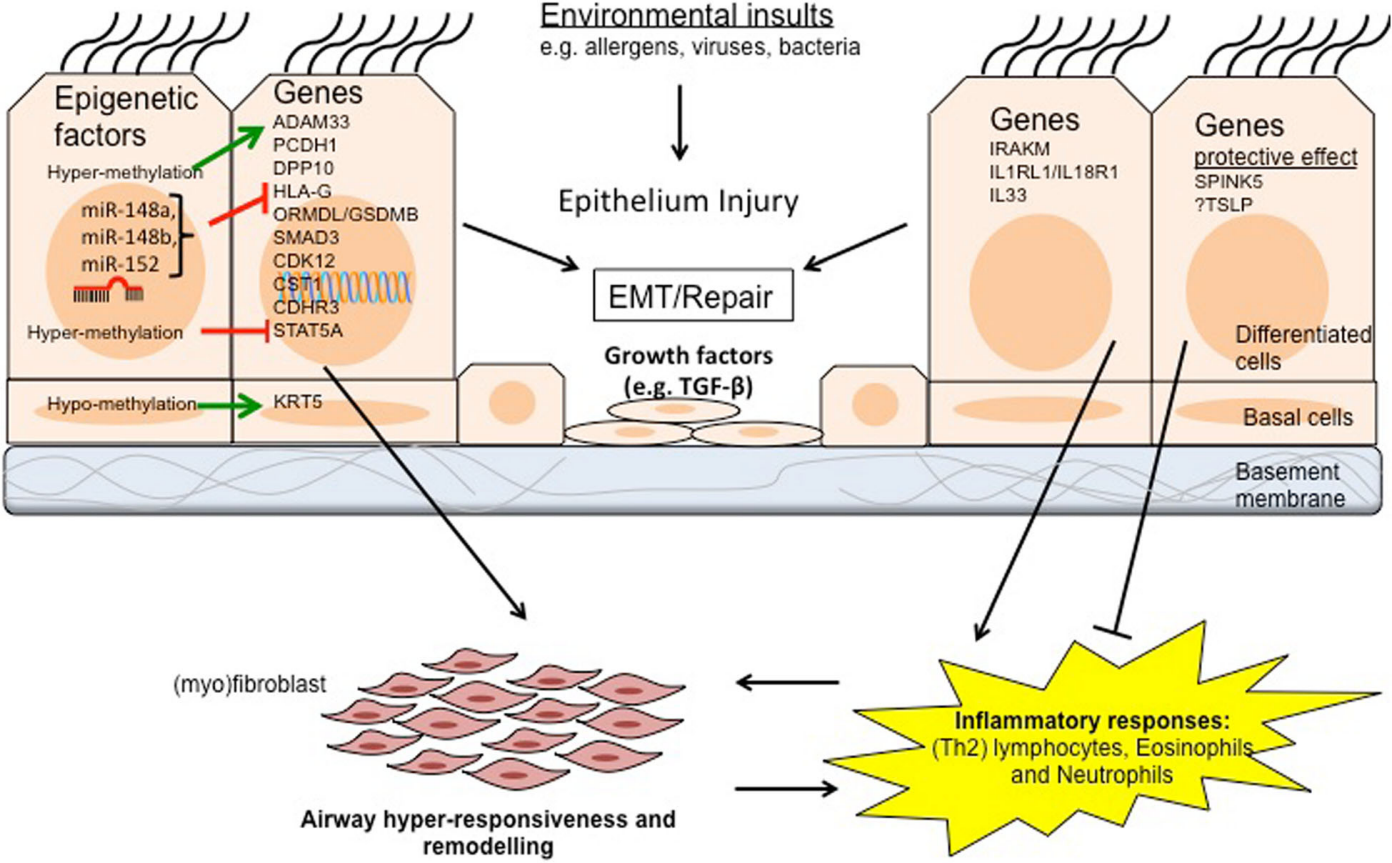
Overview of the key genes and epigenetic regulatory mechanisms associated with asthma in airway epithelial cells. Environmental insults (e.g. allergens or viruses) may damage the integrity of airway epithelial cells. Some of the susceptibility genes expressed in the airway epithelial cells (e.g. *ADAM33*) may further deteriorate this structural damage through the process of epithelial–mesenchymal trophic unit (EMTU) and hence encouraging airway remodelling and hyper-responsiveness. Whereas other asthma susceptibility genes (e.g. *IRAKM*) may promote (Th2)-immunity result in activation of inflammatory responses. Some of the genes have more protective roles (e.g. *SPINK5* and *TSLP*). Furthermore, epigenetic regulatory mechanisms may affect some of these genes (e.g. hyper-methylation of *ADAM33*, and *HLA*-*G* suppression by miRNAs)

Overall, studies on asthma susceptibility genes (Table 1) and epigenetic regulatory mechanisms (Table 2) of airway epithelial cells provide important insights in the development and progression of asthma (Fig. 3).

Among susceptibility genes detected by positional cloning (Table 1), *IRAKM* may represent a potential biomarker for early onset of asthma [26] whereas *PCDH1* may be a potential biomarker in both children and adults [22]. Although ADAM33 protein increases in epithelium of asthmatics, it is not related to severity of disease [18]. None of the genes listed above can predict specific endotypes of asthma. Some genes detected by GWAS (Table 1) have shown the potential as asthma biomarkers in a specific age group, e.g. *ORMDL3*/*GSDMB* in children [60]. However, there is little overlap between asthma susceptibility genes and their products detected with positional cloning and GWAS. Epithelial eQTL could detect more specific biomarkers for different phenotype of asthma, such as *CDHR3* associated with asthma in children with severe exacerbation [78, 85], and *CST1* [78], which can differentiate asthmatic with EIB from those with no EIB [84].

Asthma is a complex disease and it is unlikely that limited functional genes are driving the entire pathophysiological (immunological and structural) events. Other challenges for genetic assessments are neglecting information for rare variants in some populations, clinical heterogeneity of asthma and the effects of various important environmental factors including smoking, air pollution, and microbial exposures. Recent studies on epigenetic regulatory factors (Table 2) have added new insights to the field. Some of the DNA methylation signatures in epithelial cells of asthmatics may be involved in epithelial homeostasis (*KRT5* and *STAT5A*), BHR (*ADAM33*) and FeNO regulation (*ARG2*, *IL*-*6* and *iNOS*) [98–101]. Notably, *ADAM33* is also a target of methylation (Fig. 3) [101], which emphasises the importance of concomitant assessment genetic and epigenetic regulatory factors in airway epithelium of asthmatics. More profoundly, miRNA networks have been shown to regulate a major portion of post-transcriptional gene regulation [130].

There are however some challenges in interpretation of miRNAs findings from different studies. One important factor is the origin of samples; for example bronchial biopsies [137] compared with cultured primary epithelial cells [121] or bronchial epithelial brushing that may include other cells which may affect yield and type of detectable miRNAs [125]. Severity of disease [137] and varying technologies and methods of analysis may also play essential roles in outcomes of miRNAs quantifications. The most common technology used is microarray which may ignore less abundant miRNAs and may not distinguish miRNAs from other RNAs with similar sequences, such as other members of the same miRNA family [125]. quantitative PCR is an alternative, which does not necessarily measure all potentially biologically important miRNAs. Additionally, the method to analyse and present data; ΔΔCT [121, 129, 130] versus 2^-ΔΔCT^ [137], can affect the outcomes. One potential way to overcome these inconsistencies is assessing miRNA expression in primary airway epithelial cells at air liquid interface culture; a mimic of pseudostratified physiological model, using technology such as NanoString that counts individual miRNA with high accuracy.

### Future prospects of miRNA research

While a number of miRNAs have been associated with abnormalities in asthmatic epithelium and disease progression, current asthma treatments (e.g. corticosteroids) show no major effect on them [125, 130, 137]. Seeking a novel approach to target abnormally expressed miRNAs and hence restoring their normal functions may provide a novel asthma intervention strategy. Specific targeting of miRNA clusters (e.g. miR-17-92 cluster with proposed proliferative roles) may restore normal epithelial homeostasis although off-target effects should to be carefully evaluated. Further studies to identify specific miRNA-mRNA interactions and validation of target proteins important in asthma pathogenesis may provide potential steps forward to providing important insights into the development of potential intervention to reverse the epithelial abnormities in asthmatics. Additionally, manipulating epigenetic factors regulating miRNAs (e.g. re-expression of miRNAs using demethylating agents to inhibit DNA methylation of the miRNA promoter) may provide another approach to restore miRNA abnormalities in asthmatics. It is also important to clarify whether these differences in miRNAs are the major factors driving asthma or if the pathology of the disease induces these changes. Hence, additional studies in larger cohorts are essential to distinguish the effects of different asthma medications on the expression of miRNAs in bronchial epithelial cells.

Overall, there are interactions between genetic factors and epigenetic regulatory mechanisms and assessment of only one factor may not provide enough information. miRNAs expression in conjunction with other epigenetic regulatory factors may be an essential contributing factor to asthma. Understanding the mechanisms that initiate the development and progression of asthma, including regulation of gene transcription or translation, are essential to identify potential targets in airway epithelium for asthma intervention.

## Abbreviations

5mC: 5-methyl-cytosine
*ADAM33*: A disintegrin and metalloprotease 33
ALI: Air-liquid interface
*ARG2*: Arginase 2
BHR: Bronchial hyperresponsiveness
*CDHR3*: Cadherin-related family member 3
*CDK12*: Cyclin-dependent kinase 12
CNVs: Copy number variants
*CRIP1*: Cysteine-rich protein 1
*CST1*: Cystatin SN
DNMT: DNA methyltransferase
*DPP10*: Dipeptidyl-peptidase 10
dsRNA: Double-stranded RNA
ECM: Extracellular matrix
EIB: Exercise-induced bronchoconstriction
EMT: Epithelial–mesenchymal transition
EMTU: Epithelial–mesenchymal trophic unit
eQTLs: Expression quantitative trait loci
FeNO: Fractional exhalation of nitric oxide
FGF: Fibroblast growth factor
*GSDMB*: Gasdermin B
*GSTM1*: Glutathione-S-transferase M1
GWAS: Genome-wide association studies
H3K18: Histone H3 lysine 18
H3K9me3: Histone H3 lysine 9 trimethylation
HAT: Histone acetyltransferase
HDAC: Histone deacetylase
HDM: Histone demethylase
HMT: Histone methyltransferase
IFN: Interferon
*IL18R1*: IL18 receptor 1
*IL1RL1*: Interleukin (IL) 1 receptor-like 1
*iNOS*: inducible nitric oxide synthase
*IRAKM*: IL-1 receptor associated kinase-M
*KRT5*: Cytokeratin 5
LEKTI: Lympho-epithelial Kazal-type-related inhibitor
lncRNA: Long non-coding RNA
MHC: Major histocompatibility
miRNAs: microRNAs
NK: Natural killer
*NPSR1*: Neuropeptide S Receptor 1
*ORMDL3*: ORM1-like 3
*PCDH1*: Protocadherin-1
piRNA: Piwi-interacting RNA
PM10: Particulate matter with a diameter of <10 μm diameter
*PPP1R1B*: Protein phosphatase 1 regulatory subunit 1B
SNAI1: SMAD3-dependent transcription factor snail1
*SPINK5*: Serine protease inhibitor Kazal type-5
*STARD3*: StAR-related lipid transfer (*START*) Domani containing 3
*START*: StAR-related lipid transfer
*STAT5A*: Signal transducer and activator of transcription 5A
*TCAP*: Titin-cap
TGF: Transforming growth factor
Th: T-helper
TLR: Toll like receptor
*TSLP*: Thymic stromal lymphopoietin
UPR: Unfolded protein response
WGS: Whole genome sequencing
